# Coevolution signals capture the specific packing of secondary structures in protein architecture

**DOI:** 10.1007/s13238-014-0051-2

**Published:** 2014-04-05

**Authors:** Lizong Deng, Xiaoxi Dong, Aiping Wu, Tingrui Song, Taijiao Jiang

**Affiliations:** 1 Key Laboratory of Protein and Peptide Pharmaceuticals, National Laboratory of Biomacromolecules, Institute of Biophysics, Chinese Academy of Sciences, Beijing, 100101 China; 2 University of the Chinese Academy of Sciences, Beijing, 100049 China

**Dear Editor,**

Decoding protein structural characteristics and formation is essential to the understanding of its function. Despite the diversity and enormity of protein universe, the dissection of known protein structures has identified regular secondary structure elements (SSEs), namely, α-helix and β-strand, that constitute the basic structural elements of protein universe (Levitt and Chothia, 1976). As a remarkable characteristics of the overall topology of a polypeptide, the specific spatial arrangement of these SSEs is a hallmark of the protein tertiary conformation (Murzin et al., 1995).

Unraveling the rules that govern the specific packing of the SSEs is critical to understanding the folding and formation of the tertiary structure of a protein and thus its function. Previously, numerous studies have attempted to identify the factors that determine the stability and specificity of SSE associations through analysis of super-secondary structure model systems like β-sheets and coiled-coils. Using designed β-hairpins or other β-sheet model systems, researchers have identified many factors that can significantly change the folding rate, stability or strand registry of β-sheets, such as charge interactions (Smith and Regan, 1995), beta propensity (Phillips et al., 2005) and β-sheet surface complementarity (Liang et al., 2008). The coiled-coil motif is another model system for understanding of molecular mechanisms that govern the super-secondary structure formation. Through NMR study of mutated GCN4p (Matousek et al., 2007) and design of stable and specific coiled-coils (Woolfson, 2005; Oakley and Kim, 1998), researchers have suggested the importance of electrostatic and polar interactions in determining the coiled-coil packing stability and specificity. In addition, Kennan and Ryan showed that changing the length of residue side chains could dramatically influence the stability of coiled-coils (Ryan and Kennan, 2007).

Despite the extensive attention on the factors that determine the specific packing of SSEs in certain forms, most of the above discoveries were made in simple soluble model systems but not natural proteins. Thus so far a global view of the effect of these factors on the protein topology still remains unclear. In this study, we would like to provide an evolutionary perspective into the roles of physiochemical properties in spatial arrangement of SSEs in protein universe. Based on a large ortholog group dataset, we systematically analyzed the coevolution signals of a variety of physiochemical properties for association of SSEs in protein evolution.

To analyze the extent of coevolution of two SSEs with regard to different physiochemical properties during protein evolution, we designed an analysis framework, which is illustrated in Fig. S1. In our analyses, the SSEs used three-state representations, namely helix, strand and loop. For physiochemical properties, we considered charge, volume, polarity, amphiphilicity, hydrophilicity and residue conformational preference namely beta propensity. For each target protein with known structure in the ortholog database OMA (the Orthologous MAtrix project) (Schneider et al., 2007), we made sequence alignment of the target protein to its homologous proteins. Then, the SSEs of homolog sequences denoted as homolog SSEs were assigned based on the aligned regions to the SSEs in the target protein. For each target SSE (i.e. SSE A in Fig. S1), the changes in a physicochemical property during evolution can be represented as a distance vector that consists of the differed values of the physiochemical property between the homolog SSEs and target SSE (vector A in Fig. S1). The differed value of a physiochemical property between a homolog SSE and its target SSE is the averaged difference of physiochemical properties over the aligned amino-acid residues (see Methods in Supplementary for details). Finally, the Spearman’s rank correlation coefficient of the distance vectors of two target SSEs (i.e., SSE A and SSE B) was calculated as the coevolution score to indicate the extent of coevolution of the two target SSEs with regard to a physiochemical property. The design of the analysis framework presents a quantitative measure of the coevolution of SSEs in protein structures with regard to these general physiochemical properties, allowing us to understand the roles of these properties in directing the packing of SSEs during the protein folding.

To investigate how these physiochemical properties co-evolve between two SSEs, we performed a large-scale analysis on the 29014 pairs of SSEs in contact derived from the 562 non-redundant ortholog groups collected from OMA database proteins (see Methods in Supplementary for details). For each physiochemical property, the coevolution scores of SSEs in contact were compared to the correlations of shuffled distance vectors (null model) (See Methods in Supplementary for details). Fig. 1A shows the median score of SSEs in contact and random SSE associations for all SSE types regarding different physiochemical properties. Among the six physiochemical properties, the coevolution signals of four properties (charge, volume, amphiphilicity and beta propensity) showed a significant difference between SSE pairs in contact and random SSE associations (*P* < 0.01 according to Mann-Whitney *U*-test. See Table S1 for details). Notably, the coevolution signal of charge property of SSEs in contact shifts significantly to the negative score relative to the random associations (*P* < 2.2 × 10^−16^). This negative correlation indicates that the coevolution of SSE charge property follows a complementary manner, underscoring the importance of maintaining the interactions between negative and positive charges in directing secondary structure packing (Acharya et al., 2006). While for volume, amphiphilicity and beta propensity, the coevolution scores of SSEs in contact are positive and significantly higher than those of random associations (volume: *P* < 2.2 × 10^−16^, amphiphilicity: *P* < 2.2 × 10^−16^ and beta propensity: *P* < 0.01), suggesting the positively correlated changes of these properties for specific packing of SSEs during protein structure evolution.

**Figure 1:**
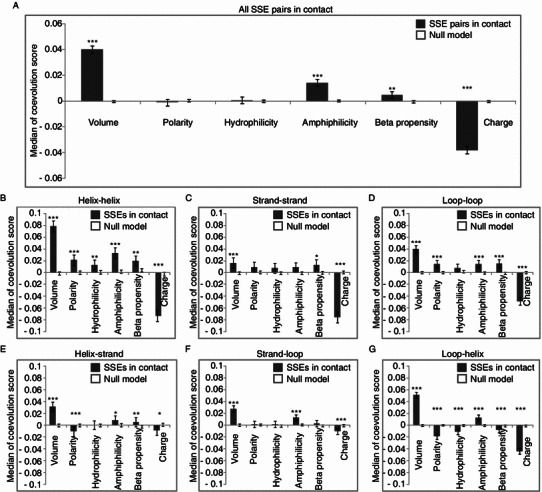
**The coevolution signals of various physicochemical properties for SSEs in contact**. (A) Comparison of coevolution signals of various properties for all types of SSE pairs in contact and random SSE associations (null model) (see Methods in Supplementary). (B–G) Comparison of coevolution signals of various properties between the SSE pairs in contact and the random SSE associations with regard to secondary structure types, for helix-helix packing (B), strand-strand packing (C), loop-loop packing (D), helix-strand packing (E), strand-loop packing (F) and loop-helix packing (G), respectively. For each property, the median values and the empirical 95% confidence intervals of coevolution scores (indicated by error bars) were plotted. *, ** and *** indicate level of significance according to Mann Whitney *U*-test: *P* < 0.05, *P* < 0.01 and *P* < 0.001, respectively

Next, we sought to examine the contribution of different physiochemical properties to the specific packing of different types of secondary structures. It was found that for all types of SSE packing, the charge and volume properties presented significant coevolution signals, suggesting that charge and volume are two key players in the formation of protein topology (Fig. 1B–G). Interestingly, it was shown that the coevolution signals of these physicochemical properties differed significantly in the packing of different secondary structure types. For example, the helix-helix packing (Fig. 1B) and loop-helix packing (Fig. 1G) had significant coevolution signals in all of the six physicochemical properties surveyed, though the strength of these coevolution signals differed. In contrast, for strand-strand packing (Fig. 1C) and strand-loop packing (Fig. 1F), except for charge and volume, there has been observed only one property with significant coevolution signal, namely beta propensity for strand-strand packing and amphiphilicity for strand-loop packing. Moreover, though the underlying properties governing loop-loop packing (Fig. 1D) and helix-strand packing (Fig. 1E) are the same, the strength of the coevolution signals for these underlying properties differed. In a word, these analyses demonstrated that different physiochemical properties contribute differently to the packing of different secondary structure types.

Since the specific spatial arrangement of SSEs is a hallmark of the topology of a protein, accurate prediction of their specific packing will greatly improve protein structure modeling. Here we would like to demonstrate whether the robust coevolution signals of SSEs in contact can improve the prediction of their specific packing. As we know, the specific pairing of β-strands is one of the most important aspects of β-sheet proteins. However, due to the weak coevolution signal of residues within β-sheet, the coevolution information has not been considered directly in predicting β-strand pairing preference (Steward and Thornton, 2002; Cheng and Baldi, 2005; Lippi and Frasconi, 2009). Given the strong coevolution signals between β-strands in contact observed in our analysis, we tested whether the consideration of such coevolution information could improve β-strand pairing prediction. To simplify our test, we integrated the coevolution scores of the six physicochemical properties computed above into a well-known β-strand pairing predictor, ΒetaPro developed by Cheng et al. (see Methods in Supplementary for details) (Cheng and Baldi, 2005). In the test, we only considered the proteins with over 200 homolog sequences. Table 1 shows when the coevolution information is considered, the precision: P = TP/(TP + FP) and recall (also known as sensitivity): R = TP/(TP + FN) are improved by 1.8% and 2.6%, respectively. The harmonic mean of precision and recall: F1 = 2PR/(P + R) is improved by 2.2%. Besides, as shown in Fig. S3, the AUC (area under the curve) of our model is 0.882, presenting a significant improvement over the original BetaPro predictor of 0.864 (*P*-value = 1.71 × 10^−4^ by Delong’s test (Delong et al. 1988)). Clearly, the incorporation of coevolution signal of β-strands can improve the prediction of their specific pairing in the formation of β-sheets, suggesting that the robust coevolutionary signals identified in our study can help us understand the molecular mechanisms underlying the formation of protein structure and thus its function.

**Table 1:** Improvement of strand pairing prediction by integrating the coevolution signals of β-strands

	Betapro	Betapro + coevolution
Precision	57.8%	59.6%
Recall	67.5%	70.1%
F1	62.3%	64.5%

10-fold cross validation was performed on the dataset of 286 proteins extracted from Cheng and Baldi dataset with more than 200 homologs. F1 is the harmonic mean of precision and recall

In summary, we carried out a systematic analysis of coevolution of six general physiochemical properties between SSEs in contact within known protein structures, and revealed robust coevolution signals of different properties for the packing of different secondary structure types. In addition, the integration of coevolution information into protein structure prediction was found to slightly improve β-strand pairing prediction. Therefore, our work could not only shed lights into the molecular mechanisms governing specific packing of SSEs in protein structure formation, but also could facilitate protein structure prediction.

## Electronic supplementary material

Below is the link to the electronic supplementary material.

Supplementary material 1 (PDF 549 kb)
