# Supplementary material for: Coevolution signals capture the specific packing of secondary structures in protein architecture
Source: Protein Cell. 2014 Apr 5;5(6):480–3. doi: 10.1007/s13238-014-0051-2 (PMC4026424; doi:10.1007/s13238-014-0051-2)
Supplement: Supplementary file 1 — Supplementary material 1 (PDF 549 kb) [file 13238_2014_51_MOESM1_ESM.pdf]

Coevolution signals capture the specific packing of secondary structures in protein architecture

Lizong Deng[1,2]*, Xiaoxi Dong[1]*, Aiping Wu[1,2], Tingrui Song[1,2], Taijiao Jiang[1 §]

[1] Key Laboratory of Protein & Peptide Pharmaceuticals, National Laboratory of Biomacromolecules, Institute of Biophysics, Chinese Academy of Sciences, Beijing 100101, China;

[2] University of the Chinese Academy of Sciences, Beijing 100049, China;

*These authors contributed equally to this work

[§]Correspondence: taijiao@moon.ibp.ac.cn or taijiao@aya.yale.edu

SUPPLEMENTAL MATERIALS:

Supplementary Methods, Discussions, References, Figures and Tables.

Materials and Methods

Dataset

Ortholog groups used for coevolution analysis were retrieved from OMA (the Orthologous MAtrix project) database of October 2009 (Schneider et al., 2007) . Only groups with at least one sequence having structure determined by X-ray diffraction were selected. For each group, if there exist over one protein with known structure, the orthologs' longest X-ray structure (PDB-target) was selected for coevolution analysis. If there exist two or more groups whose target sequences share sequence

identity above 90%, only one of them was considered. UniqueProt (Mika and Rost, 2003) with the HSSP threshold of 70 was used to remove orthologs of more than ~90% sequence identity to the target sequence in each ortholog group. After the above filtering process, 562 groups with more than 50 ortholog sequences were obtained for coevolution analysis.

β-sheet dataset were taken from Cheng and Baldi dataset (Cheng and Baldi, 2005a) . For each protein in the dataset, its homolog sequences were obtained by using PSI-BLAST (Altschul et al., 2010) to search the NCBI non-redundant database (NR). The NR database was filtered by the "coils" program to exclude coiled-coil proteins (Lupas et al., 1991). In running PSI-BLAST, E-values were set to 10E-10 in the three iterations of PSI-BLAST, and the E-value of 0.001 was used to generate position-specific profile. The profile was then used to blast against the original non-redundant database without filtering coiled-coil to generate homolog sequences using E-value cutoff 0.001. The homolog sequences with identity lower than 30% or larger than 90% to the query and those with aligned region less than 80% of the query were discarded. The remaining homolog sequences were further filtered by UniqueProt with HSSP value of 70 and aligned using MUSCLE (Edgar, 2004).

Secondary structures of ortholog sequence

For each of the 562 ortholog groups extracted from OMA dataset (see above), the structures of ortholog sequences were constructed using homology modeling based on the PDB-target of known structure (defined above) as template. Each ortholog sequence was aligned to the template using the sequence-structure alignment tool FUGUE (Shi et al., 2001). Homology-based structure modeling was performed using MODELLER (Eswar et al., 2001), and the best generated model was used as the predicted structure for the homolog sequence. Then the predicted structure was analyzed with DSSP (Kabsch and Sander, 1983) to produce secondary structure information. For simplicity, only three secondary states (helix, strand and loop) were

considered. However, the sharp turns in protein structures were identified to optimize the results of DSSP, because sharp turns often have geometries that are conformationally similar to a turn of ideal helical models and are easily identified as helix state by DSSP. Finally, A fragment of ≥ 7 amino acids with helix state was assigned as an α-helix SSE, a fragment of ≥ 4 amino acids with strand state was assigned as a β-strand SSE and a fragment of ≥ 3 amino acids with loop state was assigned as loop SSE.

For the homolog sequences obtained for the Baldi dataset, their β-strands were assigned as the aligned region to the β-strand of the proteins in the Baldi dataset in multiple sequence alignment.

Definition of SSE pairs in contact

A crucial step in our analysis is the identification of SSE pairs in contact. A pair of SSEs was considered as the candidates if two or more residues on each side were in contact. The residues were considered to be in contact if their Cα−Cα distance was below 8 Å. Only SSE pairs with a residue contact ratio (number of residues in contact divided by the total number of residues in the pair) ≥ 20% were selected for this study. In this work, totally 29014 SSE pairs in contact were derived the investigated OMA dataset, which included 2360 helix-helix packing, 3120 strand-strand packing, 4819 loop-loop packing, 3222 helix-strand packing, 8429 helix-loop packing and 7064 strand-loop packing.

Calculation of coevolution scores of different physicochemical properties between SSEs in contact

For an SSE in the PDB-target sequence (SSE_{target}), its longest aligned SSE in a homolog sequence is called a homolog SSE ($SSE_{homolog}$). The homolog SSEs ($SSE_{homolog}$) of all its homolog sequences form a homolog SSE group. When analyzing the evolution of a physiochemical property in a homolog SSE group, a

distance vector representing the distances of all $SSE_{homolog}$ to SSE_{target} with regard to a physiochemical property was computed. Six general physiochemical properties are considered in our analysis: volume, hydrophobicity, charge, betapropensity, amphiphilicity and polarity, which are described briefly in the following:

The average volume of a residue represents the average actual space that the residue occupies in protein structures, which was taken from (Pontius et al., 1996) . The polarity index of a residue measures the extent of separation of electric charge in the residue, which was taken from (Grantham, 1974). The hydrophilicity scale of a residue represents the tendency of the residue to interact with water, which was taken from (Kuhn et al., 1995). The amphiphilicity index of a residue denotes the preference of the residue to be in the interface between polar and non-polar environments, which was taken from (Mitaku et al., 2002). The beta propensity of a residue indicates its preference to appear in β-strands, which was taken from Lifson's work (Lifson and Sander, 1979). The isoelectric point of amino acid characterizes the charge of a residue, which was taken from (Zimmerman et al., 1968).

The distance vector contains a vector of property difference between the $SSE_{homolog}$ and SSE_{target}, ($A_{i,target}$) , which is the average difference between aligned residues measured by a particular physiochemical property in all non-gap positions of $SSE_{homolog}$ - SSE_{target} alignment.

$$A_{i,target} = \sum_{position\ k\ in\ SSE\ A} (I_{k,i} - I_{k,target}) / L$$

Where $I_{k,i}$ is the property index value of residue k in SSE sequence i. K is the position of residue in SSE alignment. Positions with gap are ignored. L is the number of non-gap positions in SSE alignment.

Coevolution score of two target SSEs was calculated as the Spearman's rank correlation coefficient of their corresponding distance vectors.

Construction of the null model for evaluating the significance of the coevolution

signals between SSEs in contact

The coevolution signals of packing SSEs is only meaningful with the correct construction of a null model, which is used as background signal for evaluating the significance and robustness of the observed signals. According to our methodology, the coevolution score between two SSEs in contact was calculated as the correlation coefficient between the two distance vectors (vector A and vector B in Fig. S1). The distance vector was consisted of differed values of the average physicochemical property between the homolog SSEs and target SSEs, and the correlation coefficient between the distance vectors was used to measure the strength of coordinate changes. Could the coordinated changes still exist even in random SSE associations? Therefore, we constructed the null model by randomly shuffling one of the distance vector and then calculated the distribution of correlation coefficient between the shuffled distance vectors as the null distribution.

The new pseudoenergy function of a strand pair

In order to test the contribution of the coevolution information in improving the prediction of β-sheet topology, we made the prediction by simply integrating coevolution information with Betapro (Cheng and Baldi, 2005b). In developing Betapro, Cheng and Baldi et al. first used a neural network to predict β-residue pairing probability using various sequence and evolutionary features as input. Then the residue interaction probability was used to define a pseudoenergy between any two strands, which is the highest sum of residue pairing probabilities among all possible alignments of the two strands. Finally the pseudoenergy was used in a greedy selection algorithm to predict β-strand pairing by searching for the topology that has the highest sum of pseudoenergy between all predicted pairing strands.

We integrated coevolution score and pseudoenergy from Betapro by a weighted sum of Betapro pseudoenergy and contact-vs-noncontact likelihood ratio of coevolution score measured by each property. For each property, the contact-vs-

noncontact likelihood ratio of each pair of SSEs was calculated as follows: Coevolution scores of all pairs of strands for each property were binned with binning size 0.1(See Fig. S2 for the details of distribution for contact SSE pairs and non-contact SSE pairs in each bin). For each bin B, its contact-vs-noncontact likelihood ratio C is:

C(B) =P(B |contact)/P(B |noncontact).

P(B |contact) = $N_{contact,B}$ /$N_{contact}$

Where $N_{contact,B}$ is the number of contacting pairs in bin B, $N_{contact}$ is the number of all contact pairs. Thus, P(B |contact) is the frequency of contact pairs in bin B. Similarly, P(B |noncontact) represents the frequency of noncontact pairs in bin B.

After integration, the new pseudoenergy function of a strand pair can be expressed as:

$$E = E_{betapro} + \sum_{i}^{coevolution-feature} (W_i \times C_i)$$

Where $E_{betapro}$ is the pseudoenergy calculated by Betapro. C_i is the likelihood ratio of coevolution feature i. W_i is the weighting parameter of coevolution feature i. The new pseudoenergy E is used to predict β-sheet pairing using Kruskal's algorithm to find maximum spanning tree (MST) of strands. The tree must satisfy the same three constraints as in Betapro: i) a β-residue can be partner with at most two other residues; ii) two strands pairing with the same side of the third strand cannot overlap, and iii) all strands must have at least one and at most three strand partners.

Predicting β-sheets by cross validation

The proteins in Baldi dataset (Cheng and Baldi, 2005b) that have over 200 homolog sequences were considered in prediction. There are 286 proteins having more than 200 homolog sequences. This dataset was randomly spited into 10 folds. In the 10-fold cross validation, 9 folds are used for training and 1 fold for testing.

The predictive power on test data is described by precision: P=TP/(TP+FP), recall:

R=TP/(TP+FN) and F1=2PR/(P+R). The adjustable parameters (w_i) in the pseudoenergy function were optimized using genetic algorithm by maximizing the prediction F1 on the training data.

Discussions

In this study, for a deep understanding of the molecular mechanisms governing packing of secondary structure elements (SSEs) in protein structure formation, we carried out a systematic analysis of coevolution of six general physiochemical properties between SSEs within known protein structures. The systematic analyses have revealed significantly strong coevolution signals of charge, volume, amphiphilicity and betapropensity between associated SSEs, suggesting the general roles of these properties in constraining the specific packing of SSEs in protein structure.

Charge interactions have been widely shown by experiments to have effects on protein functions. Moreover, researchers have demonstrated that charge interactions are critical in determining the specificity and stability of some super-secondary structures like β-hairpins (Schmuck et al., 2005) and coiled-coils (Matousek et al., 2007). In our analysis, we observed significant compensatory coevolution of charge property for all types of associated SSEs. This further suggests the general and important roles of ironic interactions in maintaining the stability and specificity of SSE packing in formation of native protein structure, and hence in determining the functionality of proteins.

Early studies found that the volume of hydrophobic cores of proteins are largely constant (Gerstein et al., 1994) , and the compensatory coevolution of residue volume has been hypothesized to play an important role in maintaining the compactness of protein hydrophobic core. Surprisingly, the observed coevolution signal of volume property in our study present positively correlation, rather than compensatory

coevolution. This is consistent with a previous study (Williams and Lovell, 2009), which showed that compensatory mutation of volume is not as frequent as previously thought. In their study, they did not observe many compensatory changes of volume in neighboring residues; instead, they found positive correlation between residue volume change and the contact distance change for neighboring residues. Another study also observed a positive correlation between distance of two β-strands in the formation of β-sheet and the sum of the volume of residues at their interface based on a large-scale analysis of β-sheets with known structures (Nagarajaram et al., 1999), suggesting that the volume change of neighboring β-strands could result in the change of the distance between them. Therefore, the observed positively correlated coevolution of volume property between associated SSEs may reflect a spatial flexibility in packing of these SSEs in protein structures.

Unlike the charge and volume properties, the coevolution signals of other physiochemical properties differs significantly in packing of different secondary structure types. This phenomenon actually reflects the heterogeneity of SSE packing, and the packing heterogeneity has been explored from many perspectives, such as geometric orientation (Chothia et al., 1977; Chothia and Janin, 1981; Chothia et al., 1981), packing density (Kurochkina and Privalov, 1998) and geometric complementarity (Jiang et al., 2003). For regular SSEs (α-helix and β-strand), The amino acids in these segments of secondary structure have relatively fixed conformations restricted by the constraints of the main-chain hydrogen bonds; in contrast, loops present more irregularity and flexibility compared to α-helix and β-strand. The differences of the residue composition and packing interfaces for different secondary structure types may have dominating influence on packing of different secondary structure types. However, there is a governing principle underlying the association of different secondary types, that is, packed secondary structures should have a conformation close to the minimum free-energy conformation of the isolated secondary structure (Chothia, 1984). Therefore, the

diverse properties such as residue composition and packing interfaces of secondary structure types, combining with the constraints of thermal stability, may result in the different coevolution signals underlying different types of associated SSEs.

Previously, many studies have analyzed the coevolution of residue pairs that are in contact in protein structures (Caporaso et al., 2008; Dunn et al., 2008; Yip et al., 2008). It has been shown that the constraints of residue interaction can lead to coevolution of structurally neighboring residues (Altschuh et al., 1988; Gobel et al., 1994). However, the strength of coevolution at residue level is not so evident. For example, in analysis of coevolution of residues between contact β-strands, Gregoret and Gutfreund did not observe any stronger conservation nor coevolution between pairing residues on neighboring strands than between non-contact residues on the same strand (Mandel-Gutfreund et al., 2001). This result coincides with Parisien et al's finding that the contact propensities between inter-strand residues are relatively trivial in the prediction of β-sheet topology (Parisien and Major, 2007). The coevolution of physiochemical properties at residue level has also been investigated. Fukami-Kobayashi et al found that the compensatory signal of charge in residue coevolution was relatively weak (Fukami-Kobayashi et al., 2002) . The compensatory signal of volume is even poorer. Williams et al found that among all contact amino acid pairs, only 30% demonstrate compensation for volume change, while 70% demonstrate either positively correlated changes or remain conserved (Williams and Lovell, 2009). Many factors could weaken the analysis of coevolution signals in the interacting residue pairs within a protein structure (Wollenberg and Atchley, 2000) . Particularly, it was found that residues tend to co-evolve in groups or between neighboring residues that are not necessarily in direct contact (Hamilton et al., 2004; Dutheil and Galtier, 2007; Madaoui and Guerois, 2008; Xu and Tillier, 2010). Therefore, the previous analyses could underestimate the coevolution signal between tightly packed secondary structures. In our analysis, we considered the whole secondary structures as an entity, which can take into account the group of residues

that co-evolve. Indeed, we observed strong signals of coevolution of SSEs in contact with regard to a variety of physiochemical properties during protein structure evolution. This demonstrates the advantage of our work in analysis of coevolution within protein structures. Although analysis of whole secondary structure showed significant coevolutionary signal within protein structure, there is much room for further improvement. First, a secondary structure can interact with more than one secondary structure in a protein structure, which could weaken the signals of coevolution due to ignoring the contributions from other interacting secondary structures. Second, some secondary structures make contact with each other by using only a small portion of residues, hence calculating the coevolution using whole sequence of secondary structures can bring in noises imposed by residues that are not related to packing. Third, the method we used here can be further improved by correcting for the phylogenetic noise and using distance matrix between all pairs of homolog SSEs to enlarge data set.

Supplementary References

Altschuh, D., Vernet, T., Berti, P., Moras, D., and Nagai, K. (1988). COORDINATED AMINO-ACID CHANGES IN HOMOLOGOUS PROTEIN FAMILIES. Protein Engineering 2, 193-199.

Altschul, S.F., Wootton, J.C., Zaslavsky, E., and Yu, Y.K. (2010). The Construction and Use of Log-Odds Substitution Scores for Multiple Sequence Alignment. Plos Computational Biology 6.

Caporaso, J.G., Smit, S., Easton, B.C., Hunter, L., Huttley, G.A., and Knight, R. (2008). Detecting coevolution without phylogenetic trees? Tree-ignorant metrics of coevolution perform as well as tree-aware metrics. BMC evolutionary biology 8, 327.

Cheng, J., and Baldi, P. (2005a). Three-stage prediction of protein beta-sheets by neural networks, alignments and graph algorithms. Bioinformatics 21 Suppl 1, i75-84.

Cheng, J.L., and Baldi, P. (2005b). Three-stage prediction of protein beta-sheets by neural networks, alignments and graph algorithms. Bioinformatics 21, I75-I84.

Chothia, C. (1984). Principles that determine the structure of proteins. Annual review of biochemistry 53, 537-572.

Chothia, C., and Janin, J. (1981). Relative orientation of close-packed β-pleated sheets in proteins. Proceedings of the National Academy of Sciences 78, 4146-4150.

Chothia, C., Levitt, M., and Richardson, D. (1977). Structure of proteins: packing of alpha-helices and pleated sheets. Proceedings of the National Academy of Sciences 74, 4130-4134.

Chothia, C., Levitt, M., and Richardson, D. (1981). Helix to helix packing in proteins. Journal of molecular biology 145, 215-250.

DeLong, E.R., DeLong, D.M., and Clarke-Pearson, D.L. (1988). Comparing the areas under two or more correlated receiver operating characteristic curves: a nonparametric approach. Biometrics, 837-845.

Dunn, S.D., Wahl, L.M., and Gloor, G.B. (2008). Mutual information without the influence of phylogeny or entropy dramatically improves residue contact prediction. Bioinformatics 24, 333-340.

Dutheil, J., and Galtier, N. (2007). Detecting groups of coevolving positions in a molecule: a clustering approach. BMC Evol Biol 7, 242.

Edgar, R.C. (2004). MUSCLE: Multiple sequence alignment with improved accuracy and speed. 2004 Ieee Computational Systems Bioinformatics Conference, Proceedings, 728-729

Eswar, N., Webb, B., Marti-Renom, M.A., Madhusudhan, M.S., Eramian, D., Shen, M.-y., Pieper, U., and Sali, A. (2001). Comparative Protein Structure Modeling Using MODELLER. In Current Protocols in Protein Science (John Wiley & Sons, Inc.).

Fukami-Kobayashi, K., Schreiber, D.R., and Benner, S.A. (2002). Detecting compensatory covariation signals in protein evolution using reconstructed ancestral sequences. Journal of Molecular Biology 319, 729-743.

Gerstein, M., Sonnhammer, E.L.L., and Chothia, C. (1994). Volume Changes in Protein Evolution. Journal of Molecular Biology 236, 1067-1078.

Gobel, U., Sander, C., Schneider, R., and Valencia, A. (1994). CORRELATED MUTATIONS AND RESIDUE CONTACTS IN PROTEINS. Proteins-Structure Function and Genetics 18, 309-317.

Grantham, R. (1974). Amino-Acid Difference Formula to Help Explain Protein Evolution. Science 185, 862-864.

Hamilton, N., Burrage, K., Ragan, M.A., and Huber, T. (2004). Protein contact prediction using patterns of correlation. Proteins-Structure Function and Bioinformatics 56, 679-684.

Jiang, S., Tovchigrechko, A., and Vakser, I.A. (2003). The role of geometric complementarity in secondary structure packing: a systematic docking study. Protein science 12, 1646-1651.

Kabsch, W., and Sander, C. (1983). Dictionary of protein secondary structure: pattern recognition of hydrogen‐bonded and geometrical features. Biopolymers 22, 2577-2637.

Kuhn, L.A., Swanson, C.A., Pique, M.E., Tainer, J.A., and Getzoff, E.D. (1995). Atomic and residue hydrophilicity in the context of folded protein structures. Proteins-Structure Function and Genetics 23, 536-547.

Kurochkina, N., and Privalov, G. (1998). Heterogeneity of packing: structural

approach. Protein science 7, 897-905.
Lifson, S., and Sander, C. (1979). Antiparallel and Parallel Beta-Strands Differ in Amino-Acid Residue Preferences. Nature 282, 109-111.
Lupas, A., Van Dyke, M., and Stock, J. (1991). Predicting coiled coils from protein sequences. Science 252, 1162-1164.
Madaoui, H., and Guerois, R. (2008). Coevolution at protein complex interfaces can be detected by the complementarity trace with important impact for predictive docking. Proceedings of the National Academy of Sciences of the United States of America 105, 7708-7713.
Mandel-Gutfreund, Y., Zaremba, S.M., and Gregoret, L.M. (2001). Contributions of residue pairing to beta-sheet formation: Conservation and covariation of amino acid residue pairs on antiparallel beta-strands. Journal of Molecular Biology 305, 1145-1159.
Matousek, W.M., Ciani, B., Fitch, C.A., Garcia-Moreno, B., Kammerer, R.A., and Alexandrescu, A.T. (2007). Electrostatic contributions to the stability of the GCN4 leucine zipper structure. Journal of Molecular Biology 374, 206-219.
Mika, S., and Rost, B. (2003). UniqueProt: Creating representative protein sequence sets. Nucleic Acids Res 31, 3789-3791.
Mitaku, S., Hirokawa, T., and Tsuji, T. (2002). Amphiphilicity index of polar amino acids as an aid in the characterization of amino acid preference at membrane-water interfaces. Bioinformatics 18, 608-616.
Nagarajaram, H.A., Reddy, B.V., and Blundell, T.L. (1999). Analysis and prediction of inter-strand packing distances between beta-sheets of globular proteins. Protein Eng 12, 1055-1062.
Parisien, M., and Major, F. (2007). Ranking the factors that contribute to protein beta-sheet folding. Proteins-Structure Function and Bioinformatics 68, 824-829.
Pontius, J., Richelle, J., and Wodak, S.J. (1996). Deviations from standard atomic volumes as a quality measure for protein crystal structures. Journal of Molecular Biology 264, 121-136.
Robin, X., Turck, N., Hainard, A., Tiberti, N., Lisacek, F., Sanchez, J.-C., and M üller, M. (2011). pROC: an open-source package for R and S+ to analyze and compare ROC curves. BMC bioinformatics 12, 77.
Schmuck, C., Heil, M., Scheiber, J., and Baumann, K. (2005). Charge interactions do the job: A combined statistical and combinatorial approach to finding artificial receptors for binding tetrapeptides in water. Angewandte Chemie-International Edition 44, 7208-7212.
Schneider, A., Dessimoz, C., and Gonnet, G.H. (2007). OMA Browser--exploring orthologous relations across 352 complete genomes. Bioinformatics 23, 2180-2182.
Shi, J.Y., Blundell, T.L., and Mizuguchi, K. (2001). FUGUE: Sequence-structure homology recognition using environment-specific substitution tables and structure-dependent gap penalties. Journal of Molecular Biology 310, 243-257.
Sing, T., Sander, O., Beerenwinkel, N., and Lengauer, T. (2005). ROCR: visualizing

classifier performance in R. Bioinformatics 21, 3940-3941.
Williams, S.G., and Lovell, S.C. (2009). The Effect of Sequence Evolution on Protein Structural Divergence. Molecular Biology and Evolution 26, 1055-1065.
Wollenberg, K.R., and Atchley, W.R. (2000). Separation of phylogenetic and functional associations in biological sequences by using the parametric bootstrap. Proceedings of the National Academy of Sciences of the United States of America 97, 3288-3291.
Xu, Y.B., and Tillier, E.R.M. (2010). Regional covariation and its application for predicting protein contact patches. Proteins-Structure Function and Bioinformatics 78, 548-558.
Yip, K.Y., Patel, P., Kim, P.M., Engelman, D.M., McDermott, D., and Gerstein, M. (2008). An integrated system for studying residue coevolution in proteins. Bioinformatics 24, 290-292.
Zimmerman, J., Eliezer, N., and Simha, R. (1968). The characterization of amino acid sequences in proteins by statistical methods. Journal of theoretical biology 21, 170-201.

Supplementary Figures

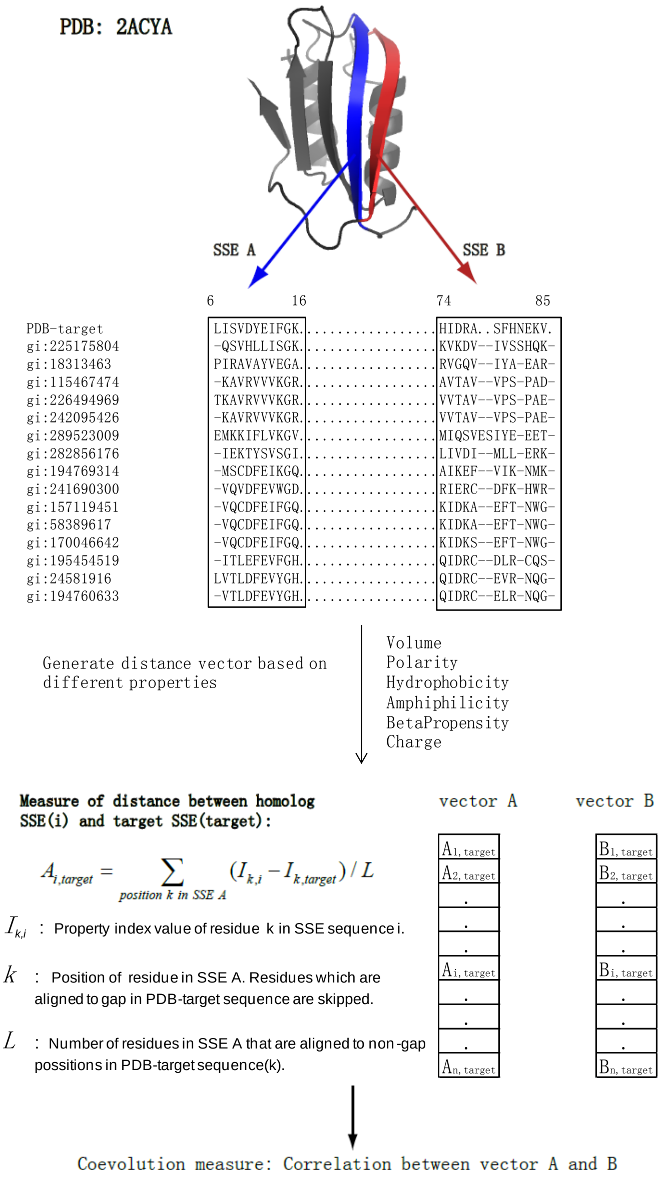

Figure S1. Algorithm diagram for analysis of coevolution between a pair of SSEs. Two secondary structure elements SSE A and SSE B are highlighted in both the structure and multiple sequence alignment. The multiple sequence alignment of the two SSEs is used to generate distance vector against target SSEs of known protein structure. For example, in SSE A, distances between ortholog SSE(i) and target SSE(target) are calculated as the average difference between residues in all non-gap positions measured by particular physiochemical property. Then the Spearman's rank

correlation coefficient of the two distance vectors is used as the measure of coevolution between SSE A and SSE B.

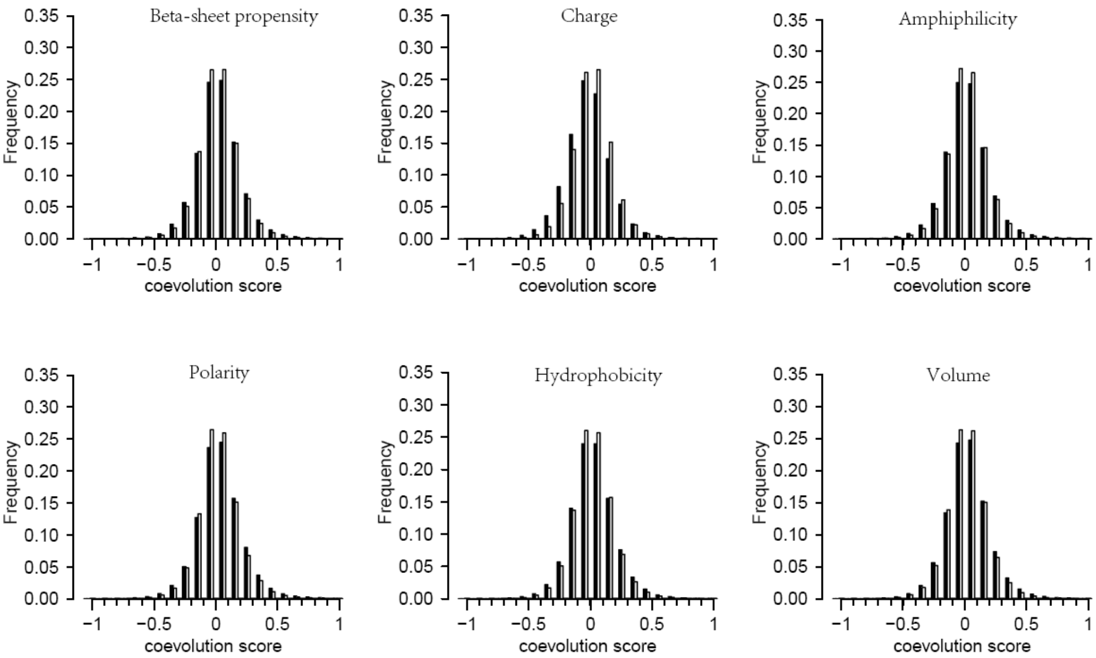

Figure S2. **The distributions of coevolution scores measured by each property for SSE pairs in contact (solid black bars) and not in contact (empty bars).**

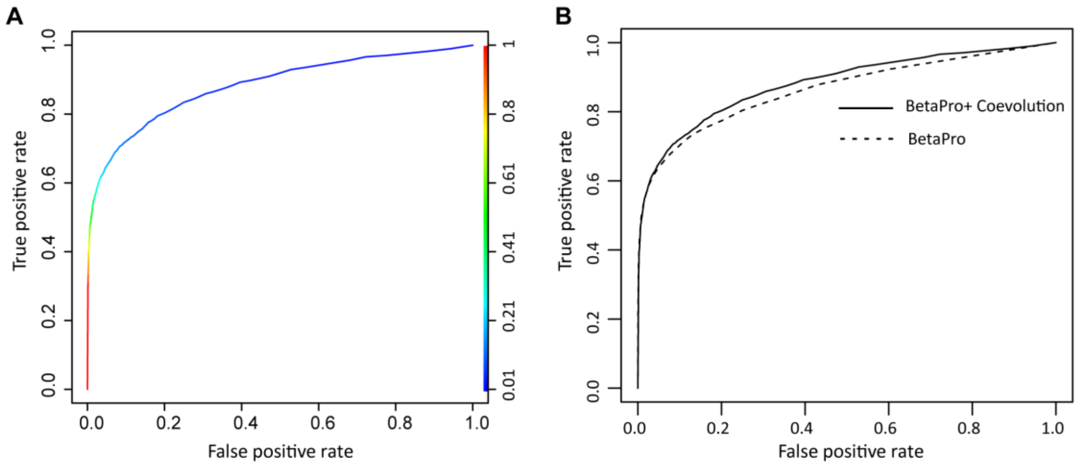

Figure S3. Comparison of the performance of β-β pairing predictions between original BetaPro predictor and the predictor integrating coevolution information. (A) ROC curve of prediction of β-β associations by integrating coevolution signals with a well-known β-strand pairing predictor (BetaPro). (B) Comparison of ROC curves between the two models: the solid line represents the ROC curve for the model which integrates coevolution signals with the pseudo-energy function developed by Betapro, and the dash line represent the ROC curve for the original BetaPro predictor. The AUC (Area Under The Curve) for the integrated model (BetaPro + coevolution information) and the original model (BetaPro) is 0.882 and 0.864 respectively, which shows significant difference according to Delong's test (DeLong et al., 1988) for two ROC curves (p-value=1.71E-04). ROC curves were plotted with ROCR package (Sing et al., 2005), and comparison of ROC curves were performed by pROC package (Robin et al., 2011).

Supplementary Tables

Table S1. The average coevolution signals of different physicochemical properties for SSE pairs in contact and random SSE associations (null model).

Property	Average Coevolution Signals		p-value
	SSEs in contact	Null Model	
volume	0.04018	-0.00013	2.20E-16
polarity	-0.0011	0.00042	6.80E-03
hydrophilicity	0.00049	-0.00003	2.39E-01
amphiphilicity	0.01409	0.00028	2.20E-16
betapropensity	0.00477	-0.0005	3.30E-03
charge	-0.0383	-0.00019	2.20E-16

Table S2. The average coevolution signals of different physicochemical properties for helix-helix packing and the corresponding significance.

Property	Average Coevolution Signals		p-value
	SSEs in contact	Null Model	
volume	0.07912	-0.00118	2.20E-16
polarity	0.02164	0.00014	1.13E-05
hydrophilicity	0.01302	0.00075	4.78E-03
amphiphilicity	0.03309	0.00207	1.11E-10
betapropensity	0.01981	0.00423	7.62E-03
charge	-0.07341	-0.00235	2.20E-16

Table S3. The average coevolution signals of different physicochemical properties for strand-strand packing and the corresponding significance.

Property	Average Coevolution Signals		p-value
	SSEs in contact	Null Model	
volume	0.01653	-0.00089	6.73E-06
polarity	0.00926	0.00049	7.51E-01
hydrophilicity	0.00789	-0.00089	1.29E-01
amphiphilicity	0.00891	-0.00083	1.18E-01
betapropensity	0.01345	-0.00061	2.89E-02
charge	-0.07548	0.00076	2.20E-16

Table S4. The average coevolution signals of different physicochemical properties for loop-loop packing and the corresponding significance.

Property	Average Coevolution Signals		p-value
	SSEs in contact	Null Model	
volume	0.04013	0.00036	2.20E-16
polarity	0.01441	0.00049	4.06E-06
hydrophilicity	0.00780	0.00182	5.44E-02
amphiphilicity	0.01489	-0.00033	7.56E-07
betapropensity	0.01583	-0.00123	1.00E-06
charge	-0.04845	0.00054	2.20E-16

Table S5. The average coevolution signals of different physicochemical properties for helix-strand packing and the corresponding significance.

Property	Average Coevolution Signals		p-value
	SSEs in contact	Null Model	
volume	0.03140	-0.00008	2.20E-16
polarity	-0.01043	0.00005	2.39E-04
hydrophilicity	0.00031	0.00019	4.21E-01
amphiphilicity	0.00840	0.00017	1.38E-02
betapropensity	0.00521	-0.00235	3.82E-03
charge	-0.00884	0.00113	4.11E-02

Table S6. The average coevolution signals of different physicochemical properties for strand-loop packing and the corresponding significance.

Property	Average Coevolution Signals		p-value
	SSEs in contact	Null Model	
volume	0.02801	0.00039	2.20E-16
polarity	0.00149	0.00068	6.77E-01
hydrophilicity	0.00131	-0.00114	0.89E-01
amphiphilicity	0.01294	0.00059	9.05E-05
betapropensity	0.00327	-0.00071	2.53E-01
charge	-0.01013	-0.00043	2.71E-06

Table S7. The average coevolution signals of different physicochemical properties for loop-helix packing and the corresponding significance.

Property	Average Coevolution Signals		p-value
	SSEs in contact	Null Model	
volume	0.05159	-0.00029	2.20E-16
polarity	-0.01876	0.00036	2.20E-16
hydrophilicity	-0.01055	-0.00015	5.10E-08
amphiphilicity	0.01297	0.00029	6.65E-09
betapropensity	-0.00789	-0.00069	1.43E-04
charge	-0.04378	-0.00067	2.20E-16
